# Measles and rubella seroprevalence among adults in Georgia in 2015: helping guide the elimination efforts

**DOI:** 10.1017/S0950268819002048

**Published:** 2019-12-11

**Authors:** N. Khetsuriani, N. Chitadze, S. Russell, M. Ben Mamou

**Affiliations:** 1 Global Immunization Division, Center for Global Health (CGH), Centers for Disease Control and Prevention (CDC), Atlanta, USA; 2 CDC South Caucasus Office, Tbilisi, Georgia; 3 National Center for Disease Control and Public Health, Ministry of Internally Displaced Persons from the Occupied Territories, Labour, Health and Social Affairs of Georgia, Tbilisi, Georgia; 4 Division of Global Health Protection, CGH, CDC, Atlanta, USA; 5 World Health Organization Regional Office for Europe, Copenhagen, Denmark

**Keywords:** Measles in Georgia, measles seroprevalence, rubella in Georgia, rubella seroprevalence

## Abstract

A large-scale measles outbreak (11 495 reported cases, 60% aged ≥15 years) occurred in Georgia during 2013–2015. A nationwide, multistage, stratified cluster serosurvey for hepatitis B and C among persons aged ≥18 years conducted in Georgia in late 2015 provided an opportunity to assess measles and rubella (MR) susceptibility after the outbreak. Residual specimens from 3125 participants aged 18–50 years were tested for Immunoglobulin G antibodies against MR using ELISA. Nationwide, 6.3% (95% CI 4.9%–7.6%) of the surveyed population were seronegative for measles and 8.6% (95% CI 7.1%–10.1%) were seronegative for rubella. Measles susceptibility was highest among 18–24 year-olds (10.1%) and declined with age to 1.2% among 45–50 year-olds (*P* < 0.01). Susceptibility to rubella was highest among 25–29 year-olds (15.3%), followed by 18–24 year-olds (11.6%) and 30–34 year-olds (10.2%), and declined to <5% among persons aged ≥35 years (*P* < 0.001). The susceptibility profiles in the present serosurvey were consistent with the epidemiology of recent MR cases and the history of the immunization programme. Measles susceptibility levels >10% among 18–24 year-olds in Georgia revealed continued risk for outbreaks among young adults. High susceptibility to rubella among 18–34 year-olds indicates a continuing risk for congenital rubella cases.

## Background

Georgia, along with other Member States of the World Health Organization (WHO) European Region, has adopted the goal for regional measles and rubella (MR) elimination [1, 2]. However, the country remains endemic for measles, while rubella virus transmission has been interrupted since 2017, as concluded by the seventh meeting of the European Regional Verification Committee for Measles and Rubella Elimination in 2018 [3].

Measles vaccine has been in use in Georgia since 1966 and rubella vaccine has been used since 2004, when measles-mumps-rubella (MMR) combination vaccine was introduced [4]. MMR is given at 12 months and 5 years of age [4]. Coverage with measles containing vaccine (one dose) was high in Georgia until the 1990s. The immunization programme in Georgia experienced serious challenges throughout the 1990s because of the collapse of the healthcare system, economic difficulties, political turmoil and armed conflicts during the first years after regaining independence in 1991 [4]. Routine coverage has improved since the mid-2000s, and has remained above 90% for MMR1 since 2010 and above 80% for MMR2 since 2013 [5]. However, the concurrent MR outbreak in Georgia during 2004–2005 resulted in more than 7000 reported cases of each disease [4]. As part of the outbreak response effort, children aged 12–14 years (born during 1990–1992) were offered MMR vaccination (coverage, 62%–86%) [4]. In 2008, a nationwide supplementary immunization activity (SIA) for MR was conducted among 6- to 27-year-olds (born during 1981–2002), but unsubstantiated vaccine safety concerns resulted in suboptimal coverage (50%) [6]. A subsequent measles outbreak during 2013–2015 resulted in 11 495 reported cases, 60% of which were among persons aged ≥15 years [7–10]. During the response to the 2013–2015 measles outbreak, contacts of measles case-patients and certain population groups (e.g. healthcare workers, military) were offered MMR vaccine. In addition, MMR vaccination has been offered free of charge to persons aged <30 years since 2013; this offer was expanded to those <40 years of age in 2018 [7–10]. Measles cases continued to be reported at a lower rate during 2016–2017 (14 cases in 2016 and 96 cases in 2017), followed by an outbreak with 2199 reported cases in 2018 (National Center for Disease Control and Public Health (NCDC), unpublished data). Twelve cases of rubella were reported in 2016 and five cases were reported in 2017. There were no reported cases in rubella in 2018.

In the past decade, many countries in the European region, including Georgia, have noted an increase in the age of MR patients [11–15]. In Georgia, this was related to immunity gaps in cohorts affected by the decline in the availability of immunization services in the 1990s (persons born during the late 1980s to mid-1990s). Older children and young adults accounted for a substantial proportion of cases in the 2004–2005 measles outbreak [4], and adults were predominantly affected in the 2013–2015 outbreak. Most laboratory-confirmed cases of rubella during 2013–2016 also occurred among adults, whereas rubella cases among children have declined substantially since 2005 [7–10].

No population-based seroprevalence surveys for MR have previously been carried out in Georgia. A nationwide population-based serosurvey to assess the prevalence of hepatitis C and hepatitis B virus infections among adults aged ≥18 years was conducted in Georgia in 2015 [16]. The availability of the residual samples from this survey provided an opportunity to assess adult population susceptibility to MR in Georgia in the immediate aftermath of the large-scale measles outbreak and during a period of low levels of rubella virus transmission. To identify and measure MR immunity gaps among adults and to help guide future interventions intended to eliminate MR, residual serum specimens from the participants of the hepatitis serosurvey were tested for Immunoglobulin G (IgG) antibodies against MR viruses.

## Methods

The original hepatitis serosurvey was based on a stratified cluster design and targeted 7000 households throughout Georgia using census enumeration areas as sampling units [16]. A total of 6011 persons aged ≥18 years were enrolled during late fall of 2015, when the measles outbreak was already over. We tested for MR IgG antibodies a subset of serosurvey participants who met all of the following criteria:

- Were aged 18–50 years.^1^
- Had consented, at the time of enrolment in the hepatitis serosurvey, to specimen storage for potential future testing.
- Had a sufficient amount of residual samples (stored at −70 °C) available for MR IgG testing.

Testing was performed under the technical guidance of the WHO Regional Office for Europe at the National Measles and Rubella Laboratory of Georgia, a WHO-accredited laboratory that is part of Georgia's National Center for Disease Control and Public Health (NCDC). The same laboratory performed testing for the original hepatitis serosurvey.

IgG antibodies against MR viruses were tested by ELISA using Enzygnost (Siemens, Germany) test kits in accordance with the manufacturer's instructions. Results with a corrected optical density (OD) value >0.2 (corresponding to 350 mIU/ml for measles antibody and 7 IU/ml for rubella antibody) were considered ‘positive’ and results with corrected OD <0.1 (corresponding to 150 mIU/ml for measles antibody and 4 IU/ml for rubella antibody) were considered ‘negative’. Specimens with corrected OD between 0.1 and 0.2 were considered ‘equivocal’ if repeated testing resulted in the OD values in the same range. For accurate representation of population susceptibility, results in the equivocal range were combined with ‘positives’ [17–19]. IgG-seronegative persons were considered ‘susceptible’ to corresponding infection.

Statistical analysis of the data was performed using SAS (version 9.4). The main outcome measure was the percentage of the population susceptible to MR. Nationwide estimates of seroprevalence were calculated for the combined group aged 18–50 years (birth cohorts of 1965–1997). Seroprevalence was also calculated by age group (18–24, 25–29, 30–34, 35–39, 40–44 and 45–50 year-old age groups^2^), sex and geographic area^3^. The complex survey design was taken into account during statistical analysis. Estimates were adjusted to account for nonresponse (nonparticipation and lack of residual samples from participants) and for individual participant's probability of selection. The data from the 2014 national census in Georgia were used for weighting the estimates. Rao–Scott *χ*^2^ tests were used to assess differences in seroprevalence across subgroups.

To examine the correlation between measles or rubella susceptibility identified by the serosurvey and disease in the same age groups, we obtained information on the epidemiology of MR in Georgia and outbreak response activities during 2013–2017 from NCDC and reports of previous investigations [6–10]. We analysed national measles-rubella surveillance data for cases from birth cohorts included in the serosurvey (1965*–*1997) and compared the proportion of reported cases by serosurvey age groups. For measles, we analysed all cases reported during the period after the serosurvey – the post-outbreak period of 2016–2017 and the outbreak year of 2018. For rubella, we analysed cases from 2013–2017, the entire period for which case-based data were available^4^, because many fewer cases were reported and year-to-year variations in incidence were not substantial. Because of concerns about the high proportion of clinically compatible rubella cases and low reliability of clinical diagnosis, a separate analysis was also done for rubella cases confirmed by laboratory testing or epidemiological linkage to laboratory-confirmed cases.

We also estimated the average number of children born annually to rubella-susceptible women during 2014–2016 based on the average number of live births by the maternal age group and the proportion of rubella-susceptibles in the serosurvey for the corresponding age group.^5^ In calculations, overall, rather than sex-specific, seroprevalence was used because of the absence of any noticeable differences by sex. The data on live births by maternal age during 2014–2016 were obtained from the National Statistics Office of Georgia [20].

CDC and NCDC determined this serosurvey, as well as the original hepatitis serosurvey, to represent public health activity (programme evaluation) and not human subject research.

## Results

A total of 3152 residual serum specimens were available for MR antibody testing. Overall, 6.3% (95% CI 4.9%–7.6%) of adults 18–50 years of age were seronegative for measles IgG antibody. Measles susceptibility declined with age from a high of 10.1% among 18–24 year-olds to 1.2% among 45–50 year-olds (*P* < 0.01). The seronegative point prevalence for the measles antibody was <10% in all age groups older than 25 years and <5% in age groups older than 40 years, although the upper 95% confidence limit exceeded those values for some age groups (Table 1). There were no significant differences in measles susceptibility by sex (*P* > 0.05). Across regions, the highest proportion of susceptibles was found in Shida Kartli (10.7%), Imereti (7.1%) and Tbilisi (6.5%). The lowest proportions were found in Samegrelo (3.4%) and Samtskhe-Javakheti (3.0%) (Table 1). Of note, 14.2% of college students (95% CI 5.4%–23.0%) were measles antibody seronegative compared with 5.7% (4.4%–7.1%) of non-students (*P* < 0.01). Only 2.0% of those who had ever served in the military (95% CI 0%–4.4%) were measles antibody seronegative, compared with 6.5% (95% CI 5.1%–7.9%) among those who had not served (*P* < 0.05) (data not shown).

**Table 1.** Susceptibility to MR among persons aged 18–50 years – Georgia, 2015

Variable	Total No. tested	IgG-negative to measles			IgG-negative to rubella		
		Crude No.	Weighted %	95% CI	Crude No.	Weighted %	95% CI
Overall	3152	180	6.3	4.9**–**7.6	257	8.6	7.1**–**10.1
Age groups (birth cohorts)							
18**–**24 years (1991–1997)	520	52	10.1	6.3**–**13.9	52	11.6	7.7**–**15.6
25**–**29 years (1986–1990)	490	42	8.0	4.8**–**11.3	77	15.3	11.1**–**19.5
30**–**34 years (1981–1985)	577	32	6.0	3.0**–**9.1	60	10.2	6.5**–**13.8
35**–**39 years (1976–1980)	501	33	7.7	2.3**–**13.1	29	4.9	2.6**–**7.1
40**–**44 years (1971–1975)	505	13	4.0	1.4**–**6.5	19	3.4	1.6**–**5.2
45**–**50 years (1965–1970)	559	8	1.2	0.4**–**2.1	20	4.4	2.1**–**6.8
Sex							
Male	1285	72	6.2	4.4**–**8.0	100	8.9	6.8**–**11.0
Female	1867	108	6.3	4.4**–**8.2	157	8.3	6.4**–**10.2
Region^a^							
Tbilisi	441	27	6.5	3.5**–**9.4	34	8.0	4.6**–**11.4
Achara	429	16	4.8	2.8**–**7.7	18	5.9	2.7**–**9.2
Imereti	494	37	7.1	3.8**–**10.4	30	7.4	4.7**–**10.1
Kakheti	399	29	5.7	1.8**–**9.7	35	4.9	2.2**–**7.6
Kvemo Kartli	238	10	5.0	0.7**–**9.4	28	14.9	8.2**–**21.6
Rustavi city	203	14	5.7	2.6**–**8.8	19	8.5	5.3**–**11.8
Samegrelo	377	15	3.4	1.6**–**5.2	35	9.4	5.1**–**13.6
Samtskhe-Javakheti	148	6	3.0	0.2**–**5.9	10	6.2	1.8**–**10.6
Shida Kartli	219	16	10.7	4.4**–**17.0	30	15.4	10.6**–**20.2

CI, confidence interval.

a Regions with <100 persons tested are excluded.

Overall, 8.6% (95% CI 7.1%–10.1%) of adults 18–50 years of age nationwide were rubella IgG antibody seronegative. Susceptibility to rubella differed significantly by the age group (*P* < 0.001). The point prevalence of IgG seronegativity for rubella was highest among persons aged 25–29 years (15.3%), followed by 18–24 year-olds (11.6%) and 30–34 year-olds (10.2%). Susceptibility to rubella declined sharply to <5% in age groups older than 35 years, but the 95% CI exceeded 5% for these age groups (Table 1). There were no significant differences by sex (*P* > 0.05). Across regions, the highest proportion of rubella susceptibility was observed in Shida Kartli (15.4%), followed by Kvemo Kartli (14.9%) and Samegrelo (9.4%). The lowest proportions were observed in Kakheti (4.9%) and Achara (5.9%) (Table 1).

**Fig. 1.**
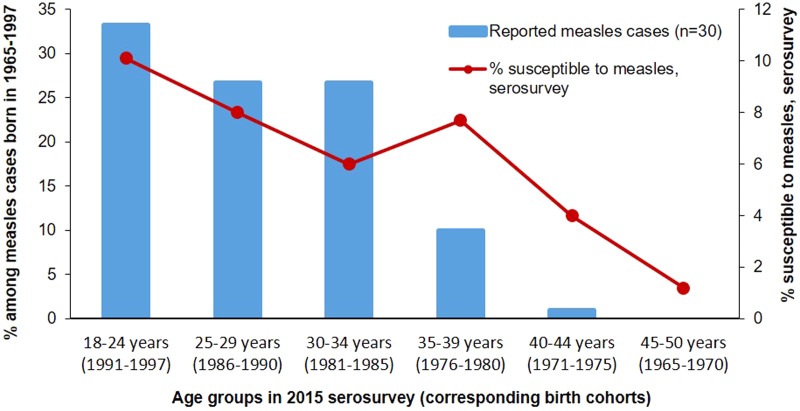
Susceptibility to measles among persons aged 18**–**50 years in 2015 and age distribution of measles cases from the same birth cohorts reported in 2016**–**2017 – Georgia.

**Fig. 2.**
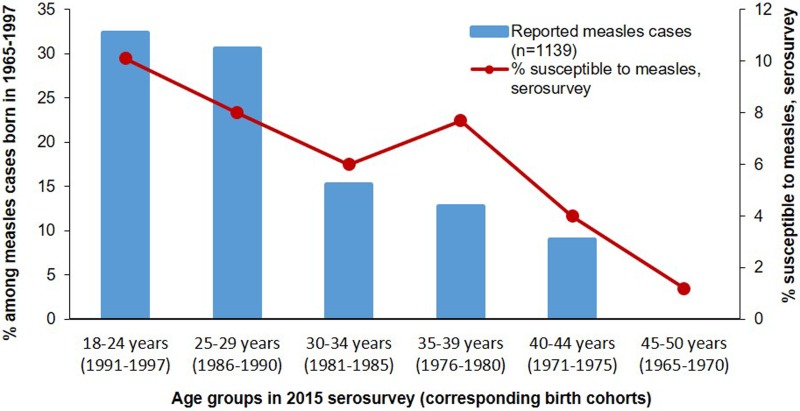
Susceptibility to measles among persons aged 18–50 years in 2015 and age distribution of measles cases from the same birth cohorts reported in 2018 – Georgia.

In the analysis of measles cases reported during 2016–2017, persons born during 1965–1997 (birth cohorts covered by the serosurvey) accounted for 30 (27.3%) of 110 cases, a substantial decline compared with 6594 (57%) of 11 495 cases reported during 2013–2015. Among these 30 measles cases, 18 (60.0%) occurred among persons 18–29 years old in 2015 at the time of the survey (1986–1997 birth cohorts), who had the highest prevalence of susceptibles in the serosurvey (Table 1, Fig. 1). Eleven (36.7%) of the reported cases during 2016–2017 occurred among those who were 30–39 years old in 2015 (1976–1985 birth cohorts). Only one (3.3%) case was reported among persons 40–50 years old in 2015 (1965–1975 birth cohorts), who had <5% point prevalence of measles susceptibility (Table 1, Fig. 1). During the 2018 outbreak of measles, the share of cases among birth cohorts covered by the serosurvey (1139 of 2199 cases, 51.8%) was higher than during 2016–2017, but lower than during the 2013–2015 outbreak. Among these 1139 cases, 717 (63.0%) occurred among persons 18–29 years old in 2015, at the time of the serosurvey (1986–1997 birth cohorts) and 319 (28.0%) – among persons 30–39 years old in 2015 (1976–1985 birth cohorts). The 1965–1975 birth cohorts (40–50 years old in 2015) which had the lowest susceptibility in the serosurvey, accounted for 103 (9.0%) of cases among surveyed birth cohorts in 2018 (Table 1, Fig. 2).

**Fig. 3.**
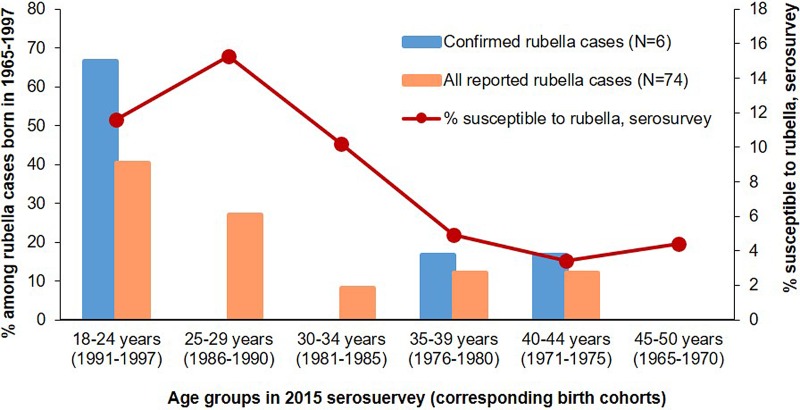
Susceptibility to rubella among persons aged 18**–**50 years in 2015 and age distribution of rubella cases from the same birth cohorts reported in 2013**–**2017 – Georgia.

In the analysis of rubella cases reported during 2013–2017, persons born during 1965–1997 (birth cohorts included in the serosurvey) accounted for only 74 (15%) of all 491 confirmed and clinically-compatible cases. Among these 74 rubella cases, persons 18–34 years old in 2015 at the time of the serosurvey (1981–1997 birth cohorts), who had the highest prevalence of rubella susceptibles in the serosurvey, accounted for 56 cases (76%), including 30 (41%) cases among 18–24 year olds (1991–1997 cohorts), 20 (27%) among 25–29 year olds (1986–1990 birth cohorts) and six (8%) cases among 30–34 year olds (1981–1985 cohorts). Persons aged 35–39 and 40–44 years old at the time of the serosurvey (1976–1980 and 1971–1975 birth cohorts, respectively) accounted for nine cases (12%) each (Table 1, Fig. 3).

Among 10 laboratory-confirmed or epidemiologically-linked rubella cases reported during 2013–2017, six (60%) occurred among birth cohorts covered by the serosurvey in 2015. Of these, four cases occurred among 18–24 years old (1991–1997 birth cohorts), who had a high prevalence of rubella susceptibility in the serosurvey. One case each occurred among persons aged 35–39 and 40–44 years in 2015 (1976–1980 and 1971–1975 birth cohorts, respectively), birth cohorts with low prevalence of susceptibility in the serosurvey (Table 1, Fig. 2).

In Georgia, on average, an estimated 6842 children (11.6% of all births) were born each year during 2014–2016 to rubella-susceptible women. Women 18 to 34 years of age accounted for the vast majority of these births (6381, or 93.2%) (Table 2).

**Table 2.** Live births by maternal age and annual average number of children born to mothers susceptible to rubella – Georgia, 2014–2016

Maternal age, years	Live births, 2014–2016			Rubella-susceptible mothers, %	Annual average no. of children born to rubella-susceptible mothers
	Total No.	% of total	Annual average No.		
15**–**17	3927	2.2	1309	11.6	152
18**–**24	63 822	36.2	21 274	11.6	2468
25**–**29	53 539	30.3	17 846	15.3	2730
30**–**34	34 796	19.7	11 599	10.2	1183
35–39	15 741	8.9	5247	4.9	257
40**–**44	3845	2.2	1282	3.4	44
45**–**50	495	0.3	165	4.4	7
>50	73	0.0	24	4.4	1
Total	176 453	100.0	58 818		6842

*Notes*. Data source on births – the National Statistics Office of Georgia (www.geostat.ge) [20]. Births among persons aged <15 years (*n* = 69) and of unknown age (*n* = 146), accounting for 0.1% of all births, are excluded. For the age groups not covered by the serosurvey, susceptibility levels in the closest age group included in the serosurvey were applied: 11.6% observed for 18–24-year-olds was applied to 15**–**17 year-olds and 4.4% observed among 45**–**50-year-olds was applied to ≥51 years-olds.

## Discussion

The serosurvey demonstrated that, in Georgia, by late 2015, >10% measles susceptibility persisted among young adults aged 18–24 years (1991–1997 birth cohort), despite their exposure to two large-scale measles outbreaks (in 2004–2005 and in 2013–2015) and the 2008 SIA. The point estimate of measles antibody seronegativity was >5% among persons up to 40 years of age (serosurvey cohorts born after 1975). These findings are consistent with the epidemiology of recent measles outbreaks that predominantly affected persons born between the mid-1980s and late 1990s, with fewer cases occurring among persons born between late 1970s and mid-1980s [4, 7]. Measles surveillance data from the post-2013–2015 outbreak period revealed a predominance of young adults during the period of relatively low incidence in 2016–2017, as well as during the subsequent outbreak. The serosurvey data also suggest that most residual susceptibility to measles among adults is concentrated in birth cohorts aged <30 years in 2015. The decline since 2013–2015 in the proportion of measles cases among persons from birth cohorts included in the serosurvey suggests certain decline in susceptibility to measles following the 2013–2015 outbreak in these cohorts. However, the serosurvey demonstrated that susceptibility levels in 2015 were sufficiently high to sustain the risk for measles outbreaks involving young adults in Georgia, as confirmed by a large outbreak in 2018. These results are consistent with 93% threshold for population immunity against measles (i.e. ≥7% seronegativity), particularly among adults, needed to prevent outbreaks [21].

The more than 10% susceptibility to measles among young adults in Georgia is concerning because of the tendency of young adults to congregate in certain occupational, social and recreational settings (non-random mixing of the population) and potential for increased transmission in these settings. In the 2013–2015 measles outbreak in Georgia, measles transmission occurred primarily within age groups; in approximately 70% of adult cases, the source of disease was another adult (NCDC, unpublished data). The preferential transmission of measles within age groups likely results from high levels of intra-group mixing in certain settings. During the 2013–2015 outbreak, occupational groups likely to involve young adults, such as college students, military recruits and police and law enforcement officials, accounted for a substantial proportion (26%) of cases with reported occupation (NCDC, unpublished data). Considering that 1 in 10 adults between the ages of 18 and 24 years and 1 in 7 college students in 2015 were susceptible to measles, the level of susceptibility, particularly in group settings where young adults predominate, was likely sufficient to support transmission and the emergence of the 2018 outbreak.

This survey showed that susceptibility to rubella was >10% among persons 18–34 years of age, despite the opportunity for rubella vaccination during the 2008 MR SIA, whereas older, unvaccinated birth cohorts appeared to be protected by natural infection. Generally, susceptibility declined with age, with the exception of lower susceptibility in the 18–24-year-old age group than among 25–29 year olds (11.6% *vs.* 15.3%). The lower susceptibility among 18–24 year olds likely resulted from the impact of the 2004–2005 rubella outbreak on these birth cohorts, in which they accounted for 47.3% of cases [4], MMR vaccination in response to this outbreak (1991–1992 cohorts; coverage, 62%–86%) [4] and moderate coverage achieved in the 2008 SIA in this age group (1991–1992 cohorts, 55%; 1993–1997 cohorts, 59%) [6]. In contrast, the 25–29 year old age group was much less affected by the 2004–2005 rubella outbreak, accounting for 8.1% of cases; had lower coverage in the 2008 MR SIA (1986–1988 cohorts, 36.9%; 1989–1990 cohorts, 55.3%); and only the 1990 cohort received MMR as part of the 2004–2005 outbreak response (coverage, 62%), resulting in higher levels of susceptibility.

Despite limitations of the quality of rubella surveillance in Georgia imposed by the small proportion of laboratory-confirmed cases (<2%), the surveillance data for 2013–2017 and the history of the rubella immunization programme in Georgia are generally consistent with serosurvey findings suggesting that young adults likely account for most of the remaining susceptibility. Likely contributors to rubella susceptibility among young adults in Georgia are the limited success of rubella immunization efforts among these birth cohorts and the absence of large-scale outbreaks since 2005. In addition, although none of the cohorts included in the serosurvey were eligible for routine rubella vaccination, the decline in rubella cases among children following rubella vaccine introduction in 2004, during the large-scale outbreak in 2004–2005, has likely limited the exposure opportunities for unvaccinated young adults and allowed the population susceptibility to persist among them. The small birth cohort (approximately 55 000–60 000) and small average household size (3.3 persons) [22] could also have limited the spread of rubella virus to young adults in Georgia.

Several attempts to close immunity gaps for MR among young adults in Georgia have been made, but consistently suboptimal coverage in these efforts [4, 6–10] has limited their impact, leading to continued susceptibility in this population. Susceptibility to rubella among young adults in Georgia indicates the potential for outbreaks involving women of childbearing age and the risk of congenital rubella syndrome (CRS), particularly given the substantial numbers of children born to rubella-susceptible mothers and the lack of functional CRS surveillance.

Younger cohorts not included in the serosurvey (from 1999 onward) have been eligible for at least one dose of rubella vaccine since MMR, recommended at 12 months and 5 years of age, was introduced into the routine childhood vaccination programme in 2004 [4]. Given the reported coverage in Georgia during 2004–2017 (between 83% and 97% for MMR1, and between 71% and 91% for MMR2) [5] and, consistent with surveillance data, these cohorts appear to be largely protected from rubella by vaccination, but ensuring protection from measles would require achieving consistently very high coverage with two doses.

To achieve elimination, MR immunity gaps among adults in Georgia must be closed. Implementing large-scale SIAs with high coverage is the WHO-recommended approach to rapidly increase population immunity to levels needed to interrupt transmission [23–28]. However, previous MR SIAs in Georgia have not been particularly successful [4, 6]. Implementing high quality large-scale mass immunization campaigns in Georgia might be even more challenging now because of the changing healthcare environment resulting in the privatization of most services, and low demand for vaccine among adults, as demonstrated by low vaccine uptake in response to recent measles outbreaks. Presently, healthcare facilities (HCF) in Georgia are private, with no defined catchment areas or populations, and offer immunization services through contracts with the state program managed by the Ministry of Health. Individuals are responsible for signing up with an HCF of their choice, but registration with an HCF is not mandatory. Providers are not motivated to vaccinate adults, nor are they used to vaccinating them, and the perceived risk of MR in the population is low. In addition, there is a high level of migration and unemployment among young adults in Georgia. All of these factors result in the lack of effective mechanisms to deliver vaccinations to young adults on a large scale. The negative experience in the 2008 SIA, derailed by unjustified vaccine safety concerns [6], is another obstacle to the willingness of the public health sector and providers to engage in large-scale mass vaccination efforts.

Seronegativity levels have not exceeding 10.1% for measles antibody and 15.3% for rubella antibody in the most susceptible cohorts, along with the existence of defined occupational groups at higher risk (e.g. college students, with 14.2% seronegativity for measles antibody), allow to consider a more feasible alternative approach to increasing population immunity – targeted immunization efforts on more limited scales. If focused on the right groups and implemented properly and in a timely manner, such efforts would help increase population immunity among adults to levels sufficient for controlling and preventing outbreaks and eventually interrupting virus transmission.

To interrupt the measles outbreaks and increase immunity to MR among the general population of young adults, the Ministry of Health offers MMR vaccination free of charge to persons up to 40 years of age. Vaccinating adults up to age 40 years, rather than age 30 years as was the case since 2013, provides the opportunity to better address rubella susceptibility among women of childbearing age, as well as among males, and would help reduce the susceptibility to measles among those aged 30–39 years to less than 5%. To increase vaccine uptake, which so far has been minimal, communication strategies targeted to the general population and healthcare providers about the availability of opportunity for MMR vaccination of adults, as well as about risks of MR and CRS, and the benefits of vaccination, should be enhanced. Particular attention should be paid to regions with the highest susceptibility identified in the present serosurvey.

Targeted vaccination of specific groups at higher risk for measles would help reduce the immunity gap in the most affected groups, also benefiting population immunity to rubella. MMR vaccination of students and persons in certain occupations (e.g. military personnel, police/law enforcement officers and first responders, as well as and HCF workers, including medical/nursing students), should be a priority.

To limit the spread of virus and prevent the emergence of future large-scale outbreaks in Georgia, ensuring high quality surveillance with vigorous investigation and early response to any measles or rubella outbreak, including prompt tracing and vaccination of susceptible contacts or population groups at high risk, identified by previous analyses or in the course of investigation, is extremely important. The quality of rubella surveillance needs further improvement. Laboratory testing of suspected cases must be ensured. To identify potential CRS cases, effective surveillance for CRS should be implemented in accordance with the national guidelines developed by NCDC^6^. In addition, screening pregnant women for rubella IgG antibodies should be encouraged to identify susceptible women and advise them to receive post-partum MMR vaccination.

The results of the present serosurvey provide insight into current levels of susceptibility to MR in the adult population of Georgia. These findings, in combination with surveillance data, results of previous outbreak investigations, historic experiences and the current healthcare landscape in Georgia, support a targeted immunization strategy among adults and enhanced surveillance, particularly for rubella/CRS, for interrupting measles virus transmission and maintaining interruption of rubella virus transmission achieved in 2017. However, to achieve and maintain elimination, efforts to increase population immunity to MR among adults should be combined with activities to further improve and sustain immunization coverage among children. Defining approaches to reducing MR susceptibility among children in Georgia was outside the scope of this report and should be addressed separately.

## Footnotes

1 Persons aged >50 years were excluded, as their vast majority could be assumed to be immune to measles and rubella.

2 Corresponding to birth cohorts of 1991*–*1997, 1986*–*1990, 1981*–*1985, 1976*–*1980, 1971*–*1975 and 1965*–*1970 years, respectively.

3 Regions with less than 100 persons tested were excluded from this analysis.

4 No rubella cases were reported in 2018.

5 Births among persons aged <15 years (*n* = 69) and by persons of unknown age (*n* = 146), accounting for 0.1% of all births, are excluded from this analysis. For the age groups not covered by the serosurvey, susceptibility levels in the closest age group included in the serosurvey were applied – 11.6% observed for 18–24 year-olds were applied to 15–17 year-olds and 4.4% observed among 45–50 year-olds to ≥51-year-olds.

6 Measles, rubella and congenital rubella syndrome surveillance and outbreak control. National public health guidelines (in Georgian) approved by Decree No. 01-215/o of the Minister of Labour, Health and Social Welfare of Georgia, 6 October 2017.
